# Assessing the toxicological interaction effects of imidacloprid, thiamethoxam, and chlorpyrifos on *Bombus terrestris* based on the combination index

**DOI:** 10.1038/s41598-022-09808-3

**Published:** 2022-04-15

**Authors:** Yongkui Zhang, Dongqiang Zeng, Lu Li, Xiuchun Hong, Hongmei Li-Byarlay, Shudong Luo

**Affiliations:** 1 Key Laboratory of Pollinating Insect Biology, Ministry of Agriculture and Rural Affairs, Institute of Apicultural Research, Chinese Academy of Agricultural Sciences, Beijing, China; 2 Guangxi Key Laboratory for Agro-Environment and Agro-Product Safety, Guangxi University, Nanning, China; 3 Western Agricultural Research Center, Chinese Academy of Agricultural Sciences, Changji, China; 4 Agricultural Research and Development Program, Department of Agriculture and Life Sciences, Central State University, 1400 Brush Row Road, Wilberforce, OH USA

**Keywords:** Environmental impact, Entomology

## Abstract

In modern agricultural production, a variety of pesticides are widely used to protect crops against pests. However, extensive residues of these pesticides in the soil, water, and pollen have negatively affected the health of nontarget organisms, especially among pollinators such as bumblebees. As an important pollinator, the bumblebee plays a vital role in agricultural production and the maintenance of ecosystem diversity. Previous research has focused on the effects of a single pesticide on pollinating insects; however, the synergistic effects of multiple agents on bumblebees have been not studied in detail. Imidacloprid, thiamethoxam, and chlorpyrifos are three of common pesticides known for severe effects on bumblebee health. It is still unknown what synergistic effects of these pesticides on pollinators. In our test, the individual and combined toxicities of chlorpyrifos, thiamethoxam, and imidacloprid to bumblebees after 48 h of oral administration were documented by the equivalent linear equation method. Our results showed that the toxicity of each single pesticide exposure, from high to low, was imidacloprid, thiamethoxam, and chlorpyrifos. All binary and ternary combinations showed synergistic or additive effects. Therefore, our research not only shows that the mixed toxicity of insecticides has a significant effect on bumblebees, but also provides scientific guidelines for assessing the safety risks to bumblebees of these three insecticide compounds. In assessing the risk to pollinating insects, the toxicity levels of laboratory experiments are much lower than the actual toxicity in the field.

## Introduction

Pollinators play significant roles in the sustainable development of ecosystems and in agricultural production^1,2^. Previous studies have suggested that pollination services depend not only on managed bees, such as honey bees^3^, but also on wild bees, such as bumblebees, leafcutter bees, and mason bees^4^. Pollinators have been reported to contribute 9.5% of the total value of human food production worldwide^5,6^. The bumblebee (*Bombus* sp.) is an indispensable wild pollinator in native plant communities throughout the temperate ecosystem^7^. Especially in recent years, with the development of artificial domestication and facility agriculture, bumblebees have been widely used as pollinators in greenhouses because of their large size, hair covering, weak phototaxis, tolerance to low temperatures, and buzz for acoustic shock pollination^8–10^. Velthuis and van Doorn reported that more than 10,000 bumblebee colonies in Europe are utilized for crop pollination per year and that the annual output value is more than 12 billion euros^11^. The pollination service of honey bees (bees) is estimated to be worth more than 15 billion U.S. dollars to American agriculture every year^12^.

However, the abundance and diversity of wild pollinators such as bumblebees and managed honey bees have been in continuous decline in some countries and regions^3,13^. Many factors are believed to be responsible for this reduction, such as the large-scale use of chemical pesticides and their metabolites^14^, parasitic infestation^2,15^, pathogenic bacterial infection^16,17^, habitat loss^18^, a lack of nutrition^19^, and climate change^20^. Among them, pesticides and their metabolites are considered the main reason for this reduction^14,21,22^. Pesticides such as imidacloprid, thiamethoxam, and chlorpyrifos are reported to negatively affect pollinator health, behavior, and their food sources^23–28^. Research evidences have shown that the survival rate of worker bumblebees decreases after exposure to 10 ng/g of imidacloprid, especially in early spring, when the bumblebees feed on food contaminated with imidacloprid and thiamethoxam, which significantly reduces their reproductive ability^22–24^. In addition, Ellis et al.^29^ found that pesticide-exposed bumblebees were more likely to die prematurely and that the surviving bees had a 46% lower final weight than control bees.

In fact, a variety of pesticide residues in the pollen and nectar may threaten the survival of pollinators. Mullin et al.^30^ discovered more than 121 different pesticides and metabolites in similar samples in North America. Different types of fungicides, herbicides, and insecticides, such as pyrethroid and neonicotinoid insecticides, have been found in pollen and beebread samples in the United States, France, the United Kingdom, Spain, Greece, and China^3,12,27,31–36^. Tong et al.^27^ detected a variety of pesticide residues in 189 pollen and 226 bee pollen samples collected in China. The pesticide with the greatest content was imidacloprid (with an average content in pollen samples of 41.9 ng/g and in beebread samples of 19.3 ng/g), thiamethoxam (with an average content in pollen samples of 44.9 ng/g and in beebread samples of 12.8 ng/g), and chlorpyrifos (with an average content in pollen samples of 49.4 ng/g and in beebread samples of 41.4 ng/g). Similarly, Wen et al*.*^28^ also found that imidacloprid and chlorpyrifos residue in the oilseed rape pollen and nectar samples. It should be pointed that the pesticide residues of imidacloprid, thiamethoxam and chlorpyrifos are frequently detected simultaneously in the pollen and nectar^27,28,33,34^. As we know, chlorpyrifos is an organophosphorus insecticide and acaricide that is widely used in agriculture and horticulture in the United States and other countries to control a wide range of foliage—and soil-borne pests on a variety of food and feed crops^27^. Imidacloprid and thiamethoxam are representatives of the first and second generation of neonicotinoid insecticides, respectively. Although the mechanism of action is same, their residues have commonly been detected extensively. Obviously, bumblebees may be negatively affected by more than one insecticide simultaneously. However, previous studies have mainly been focused on the effects of a single insecticide on bumblebees rather than the synergistic effects of several insecticides combined. Here, we tested the acute oral toxicity of three insecticides—two neonicotinoids, imidacloprid and thiamethoxam, and one organic phosphorus, chlorpyrifos—based on either individual or joint exposures as close to field conditions as possible.

## Materials and methods

### Bumblebees

Experiments were conducted between April and May 2019. Twenty-five commercial colonies of *B. terrestris* were purchased from Koppert Agricultural Co., Ltd. (Beijing, China). Each colony contained about 200 workers, a brood at all developmental stages, and a laying queen. The bumblebees were reared on a diet that included pollen and nectar and were provided by the company in an incubator with continuous darkness, at a temperature of 25 ± 1 ℃ and a relative humidity of 60 ± 10%.

### Insecticides

Chlorpyrifos (CAS No. 2921-88-2, 96% technical material(TC)) was supplied by the Hunan Research Institute of Chemical Industry (Hunan, China). Imidacloprid (CAS No. 138261-41-3, 96% TC) was supplied by Shandong Zhongnong United Biological Technology Co., Ltd. (Shandong, China). Thiamethoxam (CAS No. 153719–23-4, 97% TC) was obtained from the Hailier Pesticides and Chemicals Group (Shandong, China). Each insecticide was dissolved in dimethyl sulfoxide (DMSO) and diluted in a 50% (w/w) sugar solution as the Organization for Economic Co-operation and Development (OECD) guideline^37^ and Yue et al.^35^ described, and where the volume ratio of DMSO to sugar solution was 1:500 (v:v). The data of our preliminary experiment in this study showed that there was no significant difference between blank control and DMSO control in mortality (the average mortality for the blank control and DMSO control group is 3.33% and 2.22%, respectively, 90 workers were used for each treatment, triplicate). Each stock solution was diluted to six test concentrations by using a calibrated micropipette and volumetric flasks.

### Toxicity assessment

**Figure 1:**
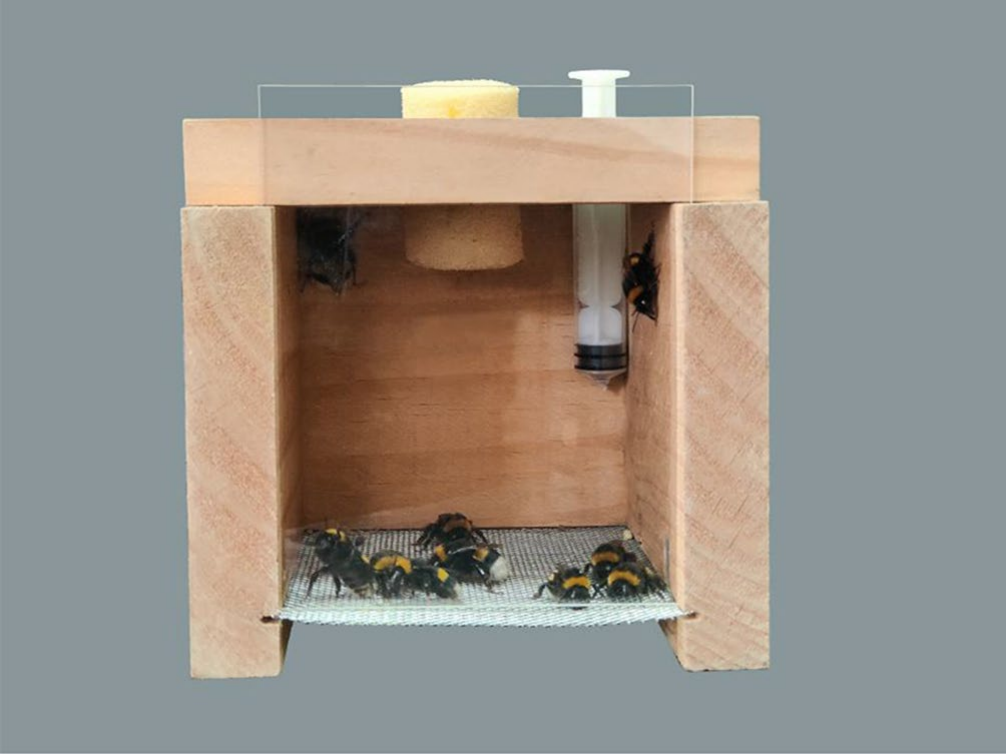
Bumblebees in the wooden for toxicity assessment.

**Figure 2:**
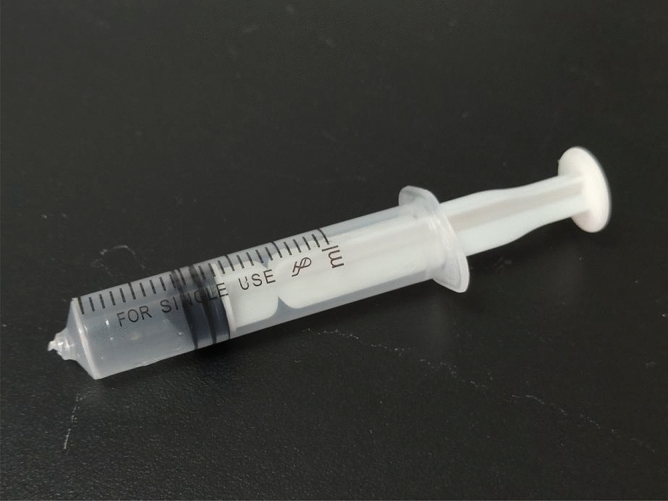
A 5 mL syringe with the tip removed.

The acute oral toxicity of the insecticides to the worker was tested according to the method recommended by the OECD^37^ (Organization for Economic Co-operation and Development). Briefly, one leg of the workers with same size was clamped gently with a forceps, and the bees were quickly transferred to a thermostat-controlled wooden box (dimensions 12 cm × 8 cm × 8 cm; Fig. 1). Fifteen workers were placed in each wooden box in the dark at room temperature (25 ± 1 ℃) and a relative humidity of 60 ± 10% with a sufficient amount of noncontaminated 50% sugar solution (w/w). The bees were left alone for at least 8 h for adaptation. The experiment was conducted when the mortality rate of bumblebees in the wooden box did not exceed 10%. A 300 μL of quantity of the 50% sugar solution was then either contaminated with an insecticide or fed uncontaminated to the worker bumblebees via a 5 mL syringe with the tip removed (Fig. 2) for 6 h, followed by 2 h of starvation. The sugar solution was immediately replaced with a sufficient amount of uncontaminated sugar solution once the 300 μL of sugar solution had been consumed over the 6 h. The mass of each test solution was weighed and recorded before and after each feeding.

All binary and ternary insecticide toxicities were administered as described by Liu et al.^36^. Stock solutions of chlorpyrifos, imidacloprid, and thiamethoxam were prepared as described above and used in three binary combinations (chlorpyrifos + imidacloprid; imidacloprid + thiamethoxam; chlorpyrifos + thiamethoxam) and a ternary combination (chlorpyrifos + imidacloprid + thiamethoxam). In total, seven treatments were performed: (1) chlorpyrifos, (2) imidacloprid, (3) thiamethoxam, (4) chlorpyrifos + imidacloprid, (5) chlorpyrifos + thiamethoxam, (6) imidacloprid + thiamethoxam, (7) chlorpyrifos + imidacloprid + thiamethoxam. The constant combination ratios were chlorpyrifos:imidacloprid, 0.568:0.310; imidacloprid:thiamethoxam, 0.310:0.438; chlorpyrifos:thiamethoxam, 0.568:0.438; and chlorpyrifos:imidacloprid:thiamethoxam, 0.568:0.310:0.438 based on the individual median lethal dose (LD_50_) toxicity such that the effects of the individual insecticides within the combination would be approximately equal. In addition, the mixed insecticides were diluted to six concentrations to perform the toxicity assessment. All the same pesticide with six different concentrations treatments were tested simultaneously to minimize experimental variations. On the other hand, six groups of workers (a total of 90 bees) from the same colony were treated with a sugar solution containing six different concentrations of pesticide treatments. Triplicate experiments were performed for one treatment, it also means that. the total number of bees used for the experiment was 1,890.

### Data analysis

A preliminary experiment suggested that evaporation of the sugar solution in the syringe did not significantly affect the mass change (a loss of about 0.001 g). Therefore, the consumption of the sugar solution could be inferred from the differences before and after insecticide exposure. The mixtures were then converted from concentrations into doses in micrograms of the active ingredient per worker. The LD_50_ values were calculated by probit analysis using POLO-PC software^38^.

The individual and combined toxic effects of insecticides on bumblebees were assessed using the median-effect equation described by Liu et al.^37^ and Chou and Talalay^39^:

1 $$f_{a} /f_{u} = \, \left( {D/D_{m} } \right)^{m}$$

where *D* is the dose of an insecticide, *D*_*m*_ is the dose for a 50% effect, *f*_*a*_ is the mortality influenced by *D* (percentage of mortality), *f*_*u*_ is the survival rate uninfluenced by *D* (percentage of survival, *f*_*u*_ = 1 − *f*_*a*_), and *m* is the coefficient determining the shape of the dose–effect relationship.

By rearranging Eq. (1), we can obtain the following equations:

2 $$f_{a} = { 1}/\left[ {{1 } + \, \left( {D_{m} /D} \right)^{m} } \right]$$

3 $$D = D_{m} [f_{a} /({1} - f_{a} )]^{{{1}/m}}$$

Therefore, if we know the values for *m* and *D*_*m*_, we can easily assess the effect (*f*_*a*_) for any given dose (*D*) in Eq. (2). In the same way, the dose (*D*) can easily be calculated by the effect (*f*_*a*_) given in Eq. (3). In addition, if we take the logarithm of both sides of Eq. (1) and assume that *x* = log(*D*) and *y* = log(*f*_*a*_/*f*_*u*_), we can obtain the following middle-effect diagram:

4 $${\text{log}}\left( {f_{a} /f_{u} } \right) \, = m{\text{log}}\left( D \right) - m{\text{log}}\left( {D_{m} } \right)$$

In the median-effect plot in Eq. (4), we can easily determine the *D*_*m*_, where *m* for the *D*_*m*_ means the antilog of the *x*-intercept and *m* is the slope. Here, *m* > 1, *m* = 1, and *m* < 1 signify sigmoidal, hyperbolic, and flat sigmoidal dose–effect curves, respectively. In addition, the linear correlation coefficient (*r*) of the median-effect plot can reveal how the data conform to the median-effect plot, where *r* = 1 shows excellent conformity.

Therefore, we can easily calculate the combination index (CI) values by using the CI equation for a combination of *n* insecticides, which is given as

5 $$n(\mathrm{CI})x={\sum }_{j=1}^{n}\frac{(D)j}{({D}_{x})j}={\sum }_{j=1}^{n}\frac{({D}_{x}{)}_{1-{\text{n}}}\{[D]j/{\sum }_{i}^{n}[D]\}}{({D}_{m}{)}_{j}\{({f}_{ax})j/[1-({f}_{ax})j{]}^{1/mj}}$$

where (CI)_*x*_ is the combination index for *n* insecticides at *x*% effect (*f*_*a*_); (*D*_*x*_)_1−*n*_ is the sum of the dose of *n* insecticides causing *x*% effect (*f*_*a*_) in combination; [*D*]*j*/${\sum }_{1}^{n}[D]$ is the proportionality of the dose of *n* individual insecticides causing *x*% effect (*f*_*a*_) in combination; (*D*_*m*_)*j*{(*f*_*ax*_)*j*/[1 − (*f*_*ax*_)*j*]^1/*mj*^} is the dose of individual insecticides causing *x*% effect (*f*_*a*_); and *f*_*ax*_ is the fractional effect (*f*_*a*_) at *x*% effect (*f*_*a*_), where CI > 1, CI < 1, and CI = 1 indicate an antagonistic, synergistic, and an additive effect, respectively.

The computer program CompuSyn^40^ was used to calculate the parameters including the dose–response curve parameters, the CI values, the *f*_*a*_–CI plot representing CI versus *f*_*a*_, the fraction influenced by a specific dose, and the polygonogram representation describing the antagonistic, additive, or synergistic effect of the insecticide combination.

## Results

### Toxicity of the three insecticides to bumblebees

All the controls had a mortality rate of 6.67% or less for acute toxicity, demonstrating the reliability of the tests. The results for each single insecticide indicated that imidacloprid had the highest toxicity (LD_50_ of 0.310 μg/bee; Table 1) among the three individual insecticide treatments. The LD_50_ of thiamethoxam was 0.438 μg/bee (Table 1), which was not significantly different from that of imidacloprid. The LD_50_ of No significant was 0.568 μg/bee (Table 1), which was significantly lower than that of imidacloprid. There was no significant difference in LD_50_ values was found between chlorpyrifos and thiamethoxam.

**Table 1:** Acute oral toxicity of pesticides (LD_50_ value) to bumblebees. Different lowercase letters in the same subcolumn indicate a significant difference among the bumblebees to different pesticide(s) (one-way ANOVA followed by Duncan’s tests). CI = combination index; C = chlorpyrifos; I = imidacloprid; T = thiamethoxam.

Pesticide(s)	Mean ± SE (µg/bee)	95% CI	
C	0.568 ± 0.123 c	0.041	1.095
I	0.310 ± 0.061 ab	0.049	0.571
T	0.438 ± 0.030 bc	0.310	0.566
C + I	0.860 ± 0.012 d	0.807	0.912
C + T	0.224 ± 0.008 a	0.190	0.257
I + T	0.205 ± 0.028 a	0.083	0.327
C + I + T	0.293 ± 0.041 ab	0.118	0.468

For the binary and ternary insecticide combinations, the two neonicotinoid insecticides (imidacloprid + thiamethoxam) were the most toxic (LD_50_ of 0.205 μg/bee; Table 1). The LD_50_ value of the binary combination of chlorpyrifos and thiamethoxam was 0.224 μg/bee (Table 1). The LD_50_ of the ternary combination of insecticides was 0.293 μg/bee (Table 1), indicating no significant difference among the combinations. Furthermore, the LD_50_ value for the binary combination of chlorpyrifos and imidacloprid (0.860 μg/bee) was significantly higher than those for the other binary and ternary insecticide combinations, indicating they had a lower toxicity.

### The combined index

The parameters *D*_*m*_, *m*, and *r* for the three neonicotinoids individually and their total combinations and the mean CI values of the total combinations are summarized in Table 2. For the individual insecticides, the *D*_*m*_ values were 0.766, 0.234, and 0.436 μg/bee for chlorpyrifos, imidacloprid, and thiamethoxam, respectively, and this result was consistent with the toxicity order of the three single insecticides after 48 h of exposure.

**Table 2:** Dose–effect relationship parameters and mean combination index (CI) values of chlorpyrifos (C), imidacloprid (I), and thiamethoxam (T) singly and in binary and ternary combinations in bumblebee tests after 48 h of exposure. The computer software CompuSyn was used to calculating the *D*_*m*_, *m*, *r*, and CI values. The parameters *D*_*m*_, *m*, and *r* are the antilog of the *x*-intercept, the slope, and the linear correlation coefficient of the median-effect plot, which indicate the potency (LD_50_), the shape of the dose–effect curve, and the conformity of the data to the mass-action law, respectively^38–40^. The *D*_*m*_ and *m* values were used to calculate the CI values (Eq. (4)), and CI < 1, CI > 1, and CI = 1 represent synergism, antagonism, and an additive effect, respectively. LD_10_, LD_50_, and LD_90_ are the doses producing a 10%, 50%, and 90% mortality rate in bumblebees, respectively. Doses are in micrograms of active ingredient per bee.

Pesticide(s)	Dose–effect parameter		CI value at		
	*D*_*m*_	*m*	LD_10_	LD_50_	LD_90_
C	0.76593	2.31045	–	–	–
I	0.23393	0.846	–	–	–
T	0.43628	5.9474	–	–	–
C + I	0.88767	4.15344	8.2139	1.76069	0.69604
C + T	0.22549	9.38724	0.599	0.37311	0.25261
I + T	0.19715	2.2892	1.68173	0.589	0.58701
C + I + T	0.31454	2.1166	1.33942	0.65273	0.70644

The antagonistic or synergistic effects were calculated based on Eq. (5) according to the *D*_*m*_ and *m* values for the single insecticides and their binary and ternary combinations^41^. The CI values at LD_10_, LD_50_, and LD_90_ indicate the doses required to produce 10%, 50%, and 90% bumblebee mortality, respectively (Table 2).

The results also indicated that the CI values at LD_10_ (8.214) and LD_50_ (1.761) for the chlorpyrifos + imidacloprid combination were greater than 1, showing a strong antagonistic effect. The same results were observed at LD_10_ (1.682) for the binary combination of imidacloprid + thiamethoxam and the ternary combination of chlorpyrifos + imidacloprid + thiamethoxam at LD_10_ (1.339). The other CI values for the combinations at each point were less than 1, indicating a strong synergy.

**Figure 3:**
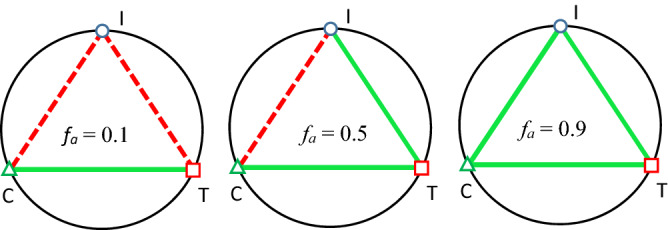
Polygonograms showing the toxicological interactions of imidacloprid (I), chlorpyrifos (C), and thiamethoxam (T) in total combinations when calculated by CompuSyn for the mortality rate of honeybees at three representative effect levels (*f*_*a*_), 0.1, 0.5, and 0.9, after an exposure for 48 h. Solid lines represent synergism, and the strength of each synergism is indicated by the thickness of the line.

**Figure 4:**
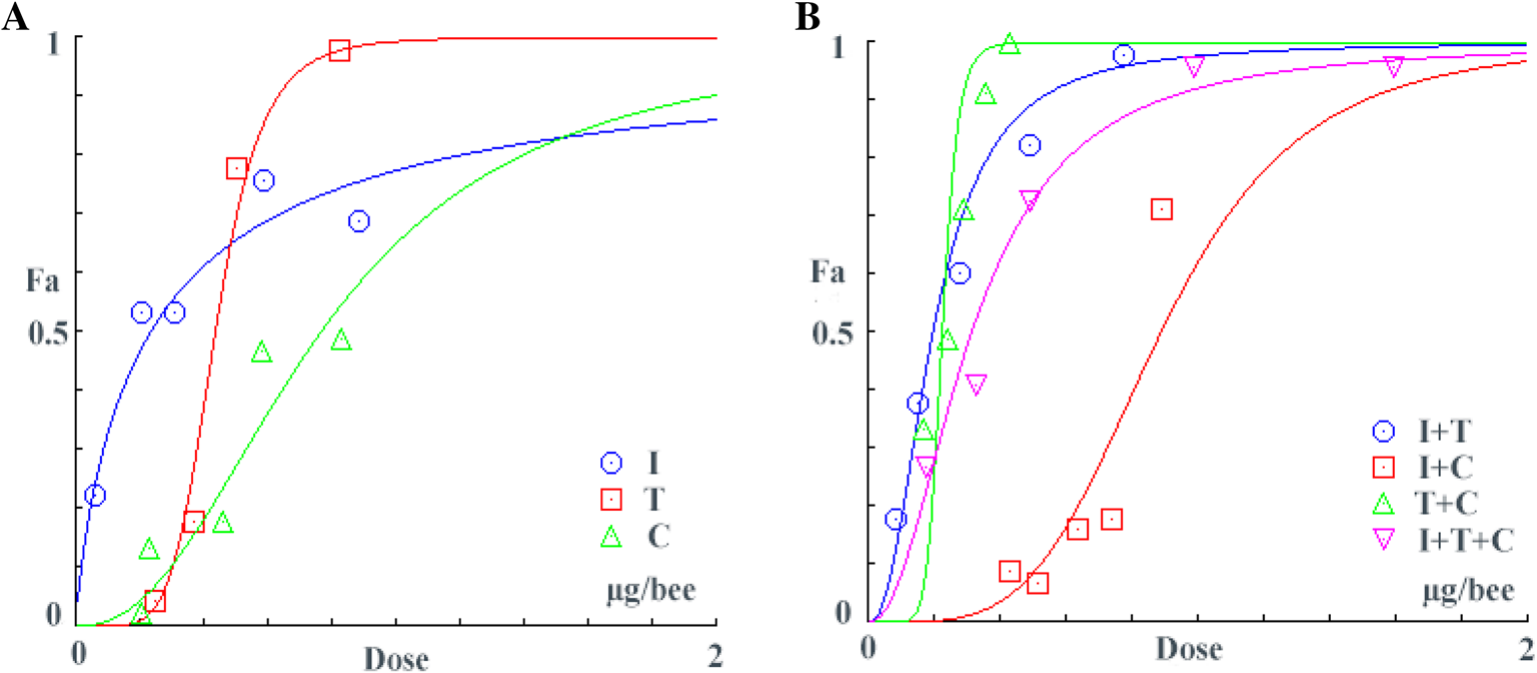
Dose–effect diagram of pesticides (**A**) and pesticide combinations (**B**). *Note* C = chlorpyrifos; I = imidacloprid; T = thiamethox.

The *f*_*a*_–CI plot can also depict the relationship between a single insecticide and a mixture of insecticides (synergistic, antagonistic, or additive effect). The computer software CompuSyn uses a semiquantitative approach to simulate a graphic for any effect (*f*_*a*_). The polygonograms revealed interactions for all the binary and ternary combinations at the 0.1, 0.5, and 0.9 effect levels after 48 h of exposure (Fig. 3). The results suggested that only chlorpyrifos + thiamethoxam had a synergistic effect at the 0.1 effect level. At the 0.5 effect level, only the combination of chlorpyrifos + imidacloprid had an antagonistic effect. All the combinations showed synergistic effects at the 0.9 effect level. These results are consistent with the CI values in Table 1. Except for imidacloprid, all the single neonicotinoids and their combinations fit the median-effect equation with an S-shaped dose–response curve (Fig. 4).

## Discussion

### Brief summary of the results

Our results showed solid measurements of LD_50_ not only in individual insecticides, but also in combinations of two or three insecticides, revealing the toxicity of the insecticide residues. Synergistic and additive effects from multiple insecticide residues were also detected, providing new evidence with which to study the toxicology of these residues in bumblebees.

Neonicotinoids were first introduced in the 1990s, and then became the most widely used class of insecticides in the world^42^. They can be found in the nectar, beebread, and honey of honey bees because of their water solubility and action as systemics^43^. Several studies have raised concerns that neonicotinoids may be having a negative effect on nontarget organisms, particularly on managed honey bees and other wild pollinators, such as bumblebees^23,44,45^. Among them, imidacloprid and thiamethoxam are found most commonly in the literature. Chlorpyrifos is one of the main organophosphates in use, and its residue has been reported in the nectar, beebread, and pollen of honey bees^27^.

The CI provided the ability to predict the joint toxicity of multiple insecticides without characterizing the insecticides according to their chemical structure and mechanism of action, as has been done previously, Such as, in an ecotoxicological evaluation of the effects of two insecticides and one herbicide on earthworms^46^, an examination of the toxicological interactions of lipid regulators in two aquatic bioluminescent organisms^47^, a study on the safety risks of three neonicotinoid mixtures to bees^38^, and an evaluation of the ecological risks of antibiotic mixtures to the aquatic environment^48^. Here, we investigated a series of interactions between two common neonicotinoids and an organophosphorus insecticide.

The results of our experiments indicated that as a single agent, imidacloprid is more toxic than chlorpyrifos or thiamethoxam. However, previous studies have shown that thiamethoxam is more toxic than imidacloprid to bees, which is exactly the opposite of our results. This disparity may be due to differences in the test insects and reagent types, given that ecotoxicity studies on different species with different nutritional levels may show completely different responses to the same toxic mixture^49^. We found that when multiple agents were mixed, as the effect gradually moved from 0 to 1, the synergy between the insecticides became more and more obvious. This finding is similar to the results of Liu et al.^38^ but differs from those of Chen et al.^46^ and Wang et al.^50^. This difference may be related to calculation of the dosage of the insecticide used and our use of the equivalent linear equation method.

In addition, except for the chlorpyrifos + thiamethoxam combination, when the effect (*f*_*a*_) was close to 0, it showed a high antagonism, and when the effect (*f*_*a*_) was close to 1, it showed a synergistic effect. The full effect (*f*_*a*_) of the binary combination of chlorpyrifos + thiamethoxam was synergistic, whereas the binary combination of chlorpyrifos + imidacloprid and the ternary combination of chlorpyrifos + imidacloprid + thiamethoxam showed an antagonistic effect. The binary combination of chlorpyrifos + imidacloprid suggested a possible competitive relationship between the two. Chlorpyrifos and imidacloprid may be combined at the same site, or they may be combined in some way and act differently at different sites. Imidacloprid and thiamethoxam are agonists of nicotinic acetylcholine receptors and can selectively bind to nicotinic acetylcholine receptors^51–53^, but Soto-Mancera et al.^54^ reported that oxypyrifos oxon, a metabolite of chlorpyrifos, can specifically inhibit nicotinic acetylcholine receptors. Whereas the combination of chlorpyrifos and imidacloprid showed an antagonistic effect, the combination of chlorpyrifos and thiamethoxam showed a synergistic effect, which may be due to the difference between the main metabolites of the two.

One reason for the interaction between mixed insecticides is that these mother fluids can be rapidly metabolized into other chemicals in insects. Previous experiments^55,56^ have shown that imidacloprid and thiamethoxam can transform various metabolites in insects and that these metabolites have very different toxicity levels to the insects. Wiesner and Kavser^56^ reported that imidacloprid was about 10 and 16 times more active against the whitefly (*Aleyrodidae*) and green peach aphid (*Myzus persicae*) than the parent imidacloprid. The activity of imidacloprid nitrosimine was similar to that of imidacloprid. N-demethylated thiamethoxam has an affinity for insect nicotinic acetylcholinerase receptors that is 1,000 times higher than that of thiamethoxam, and in insects, thiamethoxam is easily metabolized to clothianidin. Clothianidin itself belongs to the second generation of a neonicotinoid agent, which has a higher affinity for insect nicotinic acetylcholine receptors than does thiamethoxam^55,56^. Chlorpyrifos oxon, a metabolite of chlorpyrifos, can cause specific inhibition of nicotinic acetylcholine receptors.

Imidacloprid and thiamethoxam are representative of the neonicotinoid group of insecticides, which mainly block the normal conduction of the insect central nervous system by selectively controlling the nicotinic acetylcholinerase receptors in the insect nervous system, leading to paralysis and death of the insects^57,58^. Chlorpyrifos is a representative of the organophosphorus group, which destroys normal nerve activity by inhibiting the activity of acetylcholinesterase or cholinesterase^54^. In fact, because of insect resistance to a single insecticide, people have already begun using insecticide compounding to achieve high efficiency and slow the development of insect resistance. However, the specific mechanisms of compounding need to be studied further so they can be used more effectively in agricultural production and reduce the impact on nontarget organisms.

In addition, these compounding mechanisms may be the reason bumblebees take up mixed insecticides and metabolize them. As the research of Kessler et al.^59^ suggests honeybees prefer to consume sugar water containing neonicotinoid insecticides, and this preference has led to excessive intake of mixed insecticides. The absorption of one chemical insecticide will change the organism’s subsequent rate of insecticide absorption or its metabolism of other drugs, which will affect the impact of another insecticide on bumblebees. Future research is needed on the mixed effects of multiple insecticides on native pollinators such as bumblebees.

## Data Availability

All data generated or analyzed during this study are included in this article. If any additional information is on reasonable required, it may be obtained by request from the corresponding author.
